# Persistent interstitial lung abnormalities in post-COVID-19 patients: a case series

**DOI:** 10.1590/1678-9199-JVATITD-2020-0157

**Published:** 2021-04-14

**Authors:** Vanessa Carvalho Lago, Robson Aparecido Prudente, Dayane Araujo Luzia, Estefânia Thomé Franco, Talita Jacon Cezare, Amanda Peralta, Eloara Vieira M. Ferreira, André Luis Pereira Albuquerque, Marina Politi Okoshi, Bruno Guedes Baldi, Suzana Erico Tanni

**Affiliations:** 1 Department of Internal Medicine, Botucatu Medical School (FMB), São Paulo State University (UNESP), Botucatu, SP, Brazil. Department of Internal Medicine Botucatu Medical School São Paulo State University BotucatuSP Brazil; 2 Paulista School of Medicine (EPM), Federal University of São Paulo (Unifesp), São Paulo, SP, Brazil. Paulista School of Medicine Federal University of São Paulo São PauloSP Brazil; 3 Heart Institute (InCor), University of São Paulo (USP), São Paulo, SP, Brazil. University of São Paulo São PauloSP Brazil

**Keywords:** COVID-19, Interstitial lung abnormalities, CT scan

## Abstract

A new concept of multisystem disease has emerged as a long-term condition following mild-severe COVID-19 infection. The main symptoms of this affection are breathlessness, chest pain, and fatigue. We present here the clinical case of four COVID-19 patients during hospitalization and 60 days after hospital discharge. Physiological impairment of all patients was assessed by spirometry, dyspnea score, arterial blood gas, and 6-minute walk test 60 days after hospital discharge, and computed tomographic scan 90 days after discharge. All patients had fatigue, which was not related to hypoxemia or impaired spirometry values, and interstitial lung alterations, which occurred in both mechanically ventilated and non-mechanically ventilated patients. In conclusion, identifying the prevalence and patterns of permanent lung damage is paramount in preventing and treating COVID-19-induced fibrotic lung disease. Additionally, and based on our preliminary results, it will be also relevant to establish long-term outpatient programs for these individuals.

## Background

The COVID-19 pandemic has raised numerous questions on the mechanisms involved in lung injuries after mild to severe acute illness. There is great uncertainty about the possible pulmonary complications that patients with more severe manifestations may have in the longer term. We therefore present the characterization of four COVID-19 patients with persistent interstitial lung abnormalities by computed tomographic (CT) scan 90 days after hospital discharge. We also evaluated dyspnea index (baseline dyspnea index - BDI), post-bronchodilator (post-BD) spirometry, room air arterial blood gas, and 6-minute walk distance (6MWD) 60 days after discharge. The 6MWD was performed following the American Thoracic Society Guidelines [1].

The present project was approved by our local ethics committee (Botucatu Medical School, CAAE: 31258820.5.1001.5411). The Universal Trial Number (UTN) is A27072519840, the register number is RBR-8j9kqy and the public access URL is available at https://ensaiosclinicos.gov.br/rg/RBR-8j9kqy) and all patients signed the informed consent form.

Characterization of each patient is available in Table 1.

**Table 1.** Descriptive characteristics of patients and evaluation of spirometry, dyspnea score, 6-minute walk test and blood gas analysis after 60 days of hospital discharge.

	Patient 1	Patient 2	Patient 3	Patient 4
Age (y)	72	59	63	62
Gender (male/female)	Male	Male	Female	Female
Comorbidities	Ex-smoker	Hypertension Obesity Ex-smoker	Depression Hypertension Obesity Ex-smoker	Diabetes mellitus Ex-smoker
Symptom onset before hospitalization (days)	13	15	10	15
Hospitalization (days)	18	8	28	26
Maximum supplemental oxygen	Non-rebreathing mask with 15 L/min	Nasal catheter with 4 L/min	FIO_2_: 60%	FIO_2_: 90%
Mechanical ventilation (yes/no)	No	No	Yes	Yes
Antibiotics during hospitalization	Cefepime Meropenem	Ceftriaxone	Ceftriaxone Meropenem/ Vancomycin	Cefepime Meropenem/ Vancomycin
FEV1 [% (post-BD)]	110	93	89	116
FVC [% (post-BD)]	100	88	80	113
SpO_2_ (%)	96	96	95	95
BDI (score)	9	3	Not calculated	7
PaO_2_ (mmHg/kPa)	94/12.5	85/11.3	78/10.4	83/11.1
6MWD (m/% pred)	551/100	575/100	Not performed	906/178

FIO_2_: fraction of inspired oxygen, FEV1: forced expiratory volume in the first second, FVC: forced expiratory capacity, post-BD: post-bronchodilator, SpO_2_: pulse oximetry, BDI: baseline dyspnea index, PaO_2_: partial pressure of arterial oxygen, 6MWD: six-minute walk distance.

## Case presentation

### Patient 1

**Figure 1.**
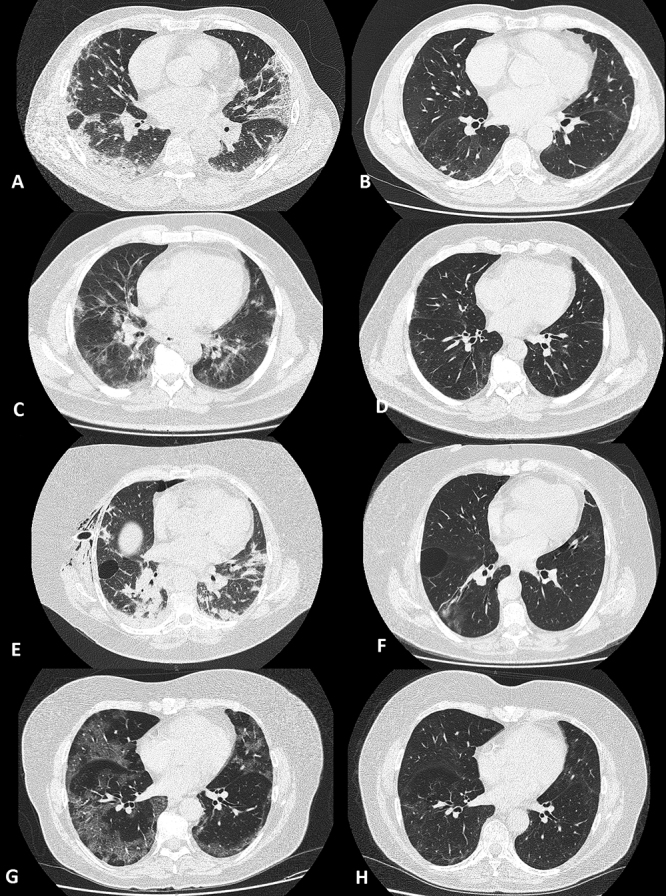
(A, C, E and G) Chest computed tomography (CT) at admission of patients 1, 2, 3 and 4, respectively. (B, D, F and H) CT 90 days after discharge of patients 1, 2, 3 and 4, respectively. All initial CT scans show bilateral, multilobar and peripheral predominance ground-glass opacities, consolidation, and septal thickening. Although all CT scans after 90 days showed a reduction in the extent and intensity of lung injury, mild ground-glass and reticular opacities with peripheral predominance remained in all patients.

A 72 year-old male ex-smoker (36 pack-years) with symptoms starting 13 days before hospitalization was discharged after 18 days. Maximal oxygen supplementation with non-rebreathing mask was 15 L/min during treatment and he was discharged without oxygen supplementation. Sixty days after discharge he reported fatigue and presented BDI 9 and normal post-BD spirometry [forced expiratory volume at the first second (FEV1) 110% of predicted value; forced vital capacity (FVC) 100% of predicted value]. Pulse oximetry (SpO_2_), partial arterial oxygen pressure (PaO_2_), and 6MWD were 96%, 94 mmHg, and 551 m (100% of predicted value), respectively [2]. CT scans at hospital admission and 90 days after discharge are presented in Figure 1 (A and B).

### Patient 2

A 59 year-old male ex-smoker (40 pack-years) with systemic arterial hypertension and obesity presented with 15 days of symptoms before hospitalization and was discharged 8 days later. Maximal oxygen supplementation with nasal prong was 4 L/min. He reported fatigue and had a BDI 3 60 days after discharge. Post-BD spirometry was normal (FEV1 93% and FVC 88% of predicted values). SpO_2_, PaO_2_, and 6MWD were 96%, 85 mmHg, and 575 m (100% of predicted value), respectively [1]. CT scans at hospital admission and 90 days after discharge are presented in Figure 1 (C and D).

### Patient 3

A 63 year-old female ex-smoker (30 pack-years) with depression, systemic arterial hypertension, and obesity with 10 days of symptoms before hospitalization was discharged 28 days later. She needed mechanical ventilation for 21 days with a maximum fraction of inspired oxygen (FIO_2_) of 60%. Sixty days after discharge she still reported severe fatigue that prevented BDI calculation and 6MWT. Post-BD spirometry was normal (FEV1 89% and FVC 80% of predicted values). SpO_2_ and PaO_2_ were 95% and 78 mmHg, respectively. CT scans at hospital admission and 90 days after discharge are presented in Figure 1 (E and F).

### Patient 4

A 62 year-old female ex-smoker (50 pack-years) with diabetes mellitus started symptoms 15 days before hospitalization and was discharged after 26 days. She needed mechanical ventilation for 15 days with a maximum FIO_2_ of 90%. The patient presented mild dyspnea with BDI 7 60 days after discharge. Post-BD spirometry was normal (FEV1 116% and FVC 113% of predicted values). SpO_2_, PaO_2_ and 6MWD were 95%, 83 mmHg and 906 m (178% of predicted value), respectively [1]. CT scans at hospital admission and 90 days after discharge are presented in Figure 1 (G and H).

## Discussion

All patients had more than 25% of pulmonary involvement at initial CT scan with a significant improvement 90 days after discharge. However, despite pulmonary function indexes within the normal range, they presented fatigue and dyspnea, and maintained interstitial lung abnormalities in follow-up CT. Acute radiological pulmonary changes caused by COVID-19 are already well described [3,4]. Chest CT scan is an essential tool for identifying viral pneumonia and classifying disease severity [4]. The most common pulmonary CT abnormalities are ground-glass opacities, consolidations, crazy-paving pattern, and linear opacities, affecting predominantly peripheral areas and lower lobes or presenting a multilobar distribution [5,6]. These images are related to interstitial edema and alveolar exudation, which can directly contribute to long-term pulmonary changes. Pulmonary CT scan is still considered as the gold standard to identify and quantify the presence of pulmonary fibrosis. In centers with difficult access to CT scan, the chest X-radiogram (X-Ray) could be used as an alternative, with a risk of twofold negative to find abnormalities in patients with COVID-19 [7]. However, the X-Ray has low sensibility to detect ground-glass opacities and lung retrocardiac alterations. In this context, the pulmonary ultrasound during the COVID-19 pandemic has been described as a potential tool during acute infection to assess the severity of the pneumonia with a good correlation with pulmonary CT scan [8,9]. However, none of the lung ultrasound studies evaluated the concordance with CT scan after the acute phase of the disease.

There are speculative questions about possible persistent pulmonary radiological abnormalities and their prevalence [7,10]. A recent follow-up of patients evaluated 15 years after severe acute respiratory distress syndrome (ARDS) showed that 4.6% presented interstitial lung changes [11]. Two of our patients were subjected to mechanical ventilation, which is a potential risk factor for pulmonary fibrosis after ARDS, that can implicate with the pulmonary repair and related to pro-fibrotic pathway. Small number of cases demonstrated that after the fourth week of symptoms onset, the pulmonary injuries can still occur in almost 30% of patients [12]. On the other hand, there is a gap of information regarding the pulmonary sequelae after long periods of follow-up [13]. Male sex is considered a risk factor for the development of severe COVID-19 [14]. However, we presented women with more severe disease.

Higher degrees of inflammatory activity are associated with greater alveolar damage; as a consequence, inflammation can be longer than virus survival [6,8]. It has been suggested that continuing respiratory symptoms after discharge can be related to persistently increased inflammatory markers. Our patients presented normal spirometry, exercise capacity and peripheral arterial blood gases, with persistent symptoms, which may be associated to increased inflammatory markers. Unfortunately, it was not possible to confirm this hypothesis as serum inflammatory markers were not assessed. Additionally, we cannot exclude the possibility that their normal functional parameters are lower than the baseline data as patients were not evaluated before the infection. 

An excessive expression of cell markers and cytokines occurs in COVID-19 [15,16]. Activation of macrophages, epithelial cells, T lymphocytes, natural killer cells (NK), and other inflammatory cells is related to increased production of proinflammatory cytokines, such as interleukin (IL)-1β, IL-6, IL-18, and tumor necrosis factor (TNF)-α, and activation of toll like receptors and the NF-κB pathway, all contributing to a cytokine storm [15,17]. Some patients have a hyperinflammatory response related to macrophages, with increased IL-1β and inflammatory markers, such as C-reactive protein, D-dimers, IL-6 and TNF-α. On the other hand, patients with inefficient activation of innate immune response may have hyperactivation and decreased number of T lymphocytes, decreased NK cells [17], and increased serum pro-inflammatory markers, especially IL-6. COVID-19 can link to CD147 of T lymphocytes that participate in cell proliferation, apoptosis, and differentiation, mainly under tissue hypoxia. The inflammatory response is thought to be related to viral antigenicity and can contribute to pulmonary fibrosis [15,18].

According to previous studies, a large release of inflammatory cytokines leads to diffuse alveolar damage in the initial phase of ARDS. The dysregulation is followed by an organizing phase, with fibrosis in connective tissue and Type II pneumocyte hyperplasia. The final stage is a fibrotic phase characterized by irreversible collagen deposition in interstitial space [17]. The factors responsible for pulmonary fibrosis development are not clear; drug-induced pulmonary toxicity, non-protective mechanical ventilation, and hyperoxia-induced damage could be involved [19]. The prevalence of COVID-19-induced pulmonary fibrosis is still unknown [20]. The increasing number of COVID-19 cases may reveal a large number of patients with long-term interstitial lung abnormalities. Recent studies have shown a decreased diffusion capacity in more than 50% of patients assessed at discharge or 30 days after infection [21,22]. Thus, we should follow COVID-19 patients to evaluate progression to irreversible fibrotic lung disease and its impact in respiratory symptoms, life quality, and mortality.

## Conclusion

In conclusion, long-term follow-up of mild-severely affected patients should also be a major focus in COVID-19 treatment. Identifying prevalence and patterns of permanent lung damage is paramount in preventing and treating COVID-19-induced fibrotic lung disease.
